# Supplementary material for: Are carbon emissions trading and green financial instruments synergistic? -Comprehensive quantitative research based on content analysis
Source: PLoS One. 2024 Mar 7;19(3):e0298601. doi: 10.1371/journal.pone.0298601 (PMC10919601; doi:10.1371/journal.pone.0298601)
Supplement: S1 Data — (ZIP) [file pone.0298601.s001.zip › Data/地级市Z_F工作报告.zip!/║■─╧/╥μ╤⌠╩╨/└┤╫╘╣1⁄2╓┌║┼í╛┬φ┐╦╩2╛▌═°í┐_2008.docx]

各位代表：

现在，我代表市人民政府，向大会作政府工作报告，请予审议，并请各位政协委员和其他列席人员提出意见。

一、2007年工作回顾

2007年，全市各级各部门坚持以邓小平理论和“三个代表”重要思想为指导，深入贯彻落实科学发展观，大力实施工业强市、项目立市、开放活市、科教兴市、依法治市五大战略，突出新型工业化和社会主义新农村建设两个重点，全市经济社会发展呈现出增长加快、效益提高、结构优化、活力增强、民生改善、社会进步的良好态势。

经济实力明显提升。预计全市完成地区生产总值408亿元，比上年增长14.8%，增幅为改革开放以来最高；人均生产总值9770元，增长14.4%。生产总值总量和人均生产总值提前实现在2000年基础上翻一番。其中第一产业增加值106亿元，增长6.5%；第二产业增加值130亿元，增长20.5%；第三产业增加值172亿元，增长15.9%。完成财政总收入25.5亿元，增长33.9%，其中地方财政收入15.3亿元，增长31.7%。实现农林牧渔业总产值173.6亿元，增长6.4%。实现工业增加值113.1亿元，增长22%。其中规模工业完成增加值86.7亿元，增长30%，实现利润6.5亿元，增长28.7%，经济效益综合指数175%，提高25.6个百分点。年末金融机构各项存款余额338.8亿元，增长14.4%，各项贷款余额192.5亿元，增长13.1%。

产业建设成效显著。新型工业化加快推进。昊华重大装备出口产业基地、晶鑫光伏产业等一批重大工业项目取得突破性进展。优势产业加速成长，特别是装备制造业增加值增长一倍，食品、新能源等产业增幅达50%以上。全市已形成以益阳高新区为龙头，区县（市）工业园和乡镇工业小区竞相发展的工业园区体系。完成高新技术产业产值49亿元，增长28%。节能减排成效显著。全市万元生产总值能耗下降4%，省政府下达的26个主要污染物削减项目全部完成。停产整治造纸企业155家、涉锑企业58家、非法炼钒企业21家、其他应淘汰企业17家，促进了资源优化配置，提升了产业素质。克明面业被评为中国驰名商标，新培育国家免检产品2个、湖南名牌12个。益阳橡塑集团获省质量管理奖。农业产业化水平居全省领先地位。全市市级以上农业产业化龙头企业达152家，其中国家级2家、省级20家。各类农民专业合作组织746个，带动农户38.3万户。农产品基地面积达到1035万亩，其中无公害、绿色和有机农产品产地认定面积385万亩，认证农产品231个。社会主义新农村示范片建设稳步推进。全市营造林96.03万亩，现代林业建设的经验在全国得到推介。黑茶产业异军突起，实现产值4亿元。市场体系不断完善，产业配套环境逐步改善。城乡市场繁荣活跃，实现社会消费品零售总额153.8亿元，增长17%。创建中国优秀旅游城市取得成效，旅游产业建设和市场开发步伐加快，接待游客人数和旅游综合收入分别增长22%、24.4%。

改革开放不断深化。国有企业改革继续推进，氮肥厂等4家市直企业完成改制，益阳麻纺厂等5家中央省属企业、轴承公司等7家市直企业启动改革。农村综合改革取得明显进展。政府收支分类改革基本到位，机关事业单位工资收入分配制度改革、国库集中收付制度改革顺利推进。调整完善了城市建设管理体制。以节能减排为目标的环境价格改革、文化体制改革和其他各项改革稳步推进。招商引资实现新的突破。全市利用市域外资金180亿元，比上年增长37.7%，其中内联引资176亿元，增长37.8%，直接利用外资5400万美元，增长30.7%。成功承办了第三届湘台经贸论坛。引进战略投资者成效显著，新引进了吉林化纤、中联重科、浙江铁牛、凯迪控股、金立集团、通威饲料、中国五矿等一批优势企业。承接沿海地区产业转移取得新进展，龙建达电子、威霖鞋业等一批产业转移项目已经投产。我市已列入全省加工贸易重点发展区。完成进出口总额2.25亿美元，增长24%，其中出口1.6亿美元，增长60%。

项目建设扎实推进。全市在建项目1239个，比上年增加142个，其中亿元以上的大型项目98个，新开工项目706个。完成全社会固定资产投资195亿元，增长51.2%，其中130个重点调度项目完成投资80亿元，增长25%。完成交通基础设施建设投资11.12亿元，沅茅公路、马奎公路建成通车，南茅复线公路、益芦航道等项目正在抓紧建设。益阳电厂二期工程进度加快，3号机组建成投入使用，资水梯级电站建设进展顺利。城乡电网建设完成投资4.4亿元。中心城区城市防洪工程完成投资5000万元，新建了高新大道、迎宾东路、梓山西路等10条城市道路，十洲路、银城南路等续建项目顺利推进，益宁城际干道正式开工。全市完成小城镇建设投资8.5亿元，增长13.3%。建筑业和房地产业迅速发展，完成房地产投资24.2亿元，增长1.4倍。防汛抗旱工作全面加强，山洪地质灾害防御成效显著。

社会事业全面进步。科技创新体系建设进度加快，建立留学生创业园和国家级企业技术中心各1个，建成省级企业（工程）技术中心和共性技术服务平台6个。国家家用杀虫用品质量监督检测中心落户益阳。与国防科大、中南大学等6所高校和科研院所签订了全面战略合作协议。农业科技服务体系逐步完善。35个科普示范村、11个科普示范社区通过验收。全市共申请专利240项，增长26%。各级各类教育稳步发展，国家“两基”督导检查顺利通过，农村义务教育经费保障机制初步建立。“名师工程”全面启动，素质教育进一步推进。中心城区教育资源整合成效显著。申办益阳医专取得成功。全市高考录取率达63.2%，高于全省8.3个百分点。卫生事业加快发展，基层卫生服务网络建设进一步加强，有效控制了各类重大传染病疫情的发生，血吸虫病疫区改厕项目全面完成。计划生育工作全面加强，市级实现进位争先目标。市博物馆完成主体工程建设。成功举办了国际正义人士何凤山博士逝世十周年纪念活动。安化千两茶、益阳茯砖茶成功申报国家级非物质文化遗产项目。文艺创作和群众文艺进一步繁荣。广播电视综合覆盖率达到98%，有线网络整合取得实质性进展。与香港凤凰卫视联合摄制了《纵横中国？城市故事益阳篇》。《益阳市志》顺利通过省级专家评审，地方志工作获得全国先进。成功举行了全省第一站“迎奥五环潇湘行”万人健步走活动。我市运动员参加第十二届特奥会夺得11枚金牌，参加全省青少年常年比赛获得33枚金牌。环境保护力度加大，城市环境质量保持全省领先地位。加强了耕地、林地、矿产资源保护和土地市场管理。关爱留守儿童工作扎实推进，妇女儿童合法权益得到保障。其他各项事业均有新的进步。

人民生活继续改善。全市城镇居民人均可支配收入10800元，实际增长12.4%；农民人均纯收入3850元，实际增长9%。就业和社会保障工作全面加强。扶贫开发继续推进。全市15座大中型水库登记农村移民15.12万人，共拨付后期扶持资金7800万元。民政和残疾人工作成效明显。我市为民办8件实事任务全面完成，共投入资金8.5亿元。全市新增城镇就业4.79万人，实现城镇零就业家庭就业援助850户；新增企业基本养老保险参保人数1.65万人，城市低保确保了按月足额发放，农村低保全面启动。完成农村公路建设里程2285公里，麻河口大桥建成通车，赤山大桥主桥基本建成。改扩建乡镇卫生院7所、敬老院17所，新型农村合作医疗总参合率达77.6%。城市污水处理率达到65.7%，垃圾无害化处理率达到100%，新建廉租房2.84万平方米。解决了10.32万农村人口的饮水安全问题。新建沼气池15718个。“乡乡能上网”、广播电视“村村通”工程年度任务全面完成。农村义务教育阶段免除学杂费、部分计划生育家庭奖励扶助政策全面落实。

社会大局保持稳定。切实强化维护稳定责任制，加强社会治安综合治理，开展平安创建活动，加大信访问题和涉法涉诉等问题的处理力度，加强矛盾纠纷的排查调处，努力防范和稳妥处置各类突发性、群体性事件，严厉打击黑恶势力、“两抢一盗”、吸毒贩毒等违法犯罪行为，深入开展“打彩禁赌”、“扫黄打非”等专项行动，社会大局保持稳定。强化安全监管，安全生产形势进一步稳定。开展产品质量和食品、药品安全专项整治，努力平抑市场物价，依法打击非法传销和制假售假行为，规范了市场经济秩序。

精神文明和民主法制建设得到加强。掀起学习贯彻党的十七大精神的热潮，加强了落实科学发展观与和谐文化建设的教育与宣传。实施了以“树立荣辱观、践行益阳精神”为主题的公民道德建设工程。弘扬文明新风，广泛开展了群众性精神文明创建活动。认真实施监督法，严格执行人大及其常委会的决议决定，自觉接受人大的法律监督、工作监督和政协的民主监督，广泛听取各方面的意见建议，决策办事机制逐步完善。办理省、市人大代表建议113件，市人大代表议案1件，省、市政协委员提案183件，办结率100%。积极推进全民国防教育，国防动员、后备力量建设和人防工作进一步加强。依法治市战略继续推进，普法教育和政府法制工作取得新成效。村（居）民自治和民主管理制度不断完善，厂务公开工作获得全国先进。大力开展以“优化政务环境、创群众满意机关”为主题的作风建设活动，全面推行有错无为问责制，建立以绩效考核为重点的考核机制，进一步规范行政审批和收费行为，切实强化审计监督职能，加大行政效能监察力度，坚持不懈开展纠风治乱和推进反腐败斗争，政府自身建设得到加强。

各位代表，过去的一年，是我市经济提速提质、社会和谐进步的一年，国民经济和社会发展取得了丰硕成果。这些成绩的取得，得益于市委的正确领导，得益于市人大、市政协的支持和监督，得益于社会各界的关心和帮助，是全市各级各部门和全市人民团结奋斗、扎实工作的结果。在此，我代表市人民政府，向全市广大干部群众，向驻益人民解放军、武警、民兵预备役官兵和政法干警，向所有关心支持益阳发展的各级领导、各界人士，表示衷心的感谢并致以崇高的敬意！

在看到成绩的同时，也要清醒地认识到，我们的工作与人民群众的要求还有差距，发展中还面临不少的困难和问题。虽然发展速度加快，但经济总量仍然较小；工业基础薄弱，经济发展方式转变不快；农民持续增收困难较大；劳动就业、社会保障、教育卫生、居民住房、安全生产、环境保护、社会治安等关系民生和影响社会稳定的问题仍然较多；经济发展环境有待进一步改善，政府自身建设需要进一步加强。这些问题值得我们高度重视，认真加以解决。

二、2008年工作的指导思想和奋斗目标

2008年，是全面贯彻落实党的十七大作出的战略部署的第一年，我们将迎来改革开放30周年。世界经济有望继续保持平稳增长，国际产业转移和技术转移进一步加快，跨国投资并购活动更加活跃。中央进一步完善和加强宏观调控，实施稳健的财政政策和从紧的货币政策，国内经济将持续平稳较快增长；国家加快实施中部地区崛起战略，将进一步加大对中西部的支持力度。从省内来看，“一化三基”战略的实施，国家批准长株潭作为资源节约型、环境友好型社会建设的综合改革试验区，为全省经济发展增强了动力与活力。我市经济发展已经进入快车道，蓄积的发展能量开始迸发，后发赶超的态势初步显现。全市干部群众思想更加统一，目标更加明确，信心更加坚定，加快发展劲头更足。我们一定要按照市第四次党代会确定的发展思路，抢抓机遇，振奋精神，开拓进取，扎实工作，努力加快后发赶超步伐。

2008年政府工作的指导思想是：全面贯彻党的十七大精神，高举中国特色社会主义伟大旗帜，以邓小平理论和“三个代表”重要思想为指导，深入贯彻落实科学发展观，紧紧围绕“十一五”期间我市综合实力跻身全省第二方阵的奋斗目标，进一步解放思想，大力推进改革开放，大力转变发展方式，大力推进新型工业化、新型城市化和社会主义新农村建设，突出抓好招商引资和项目建设，着力保障和改善民生，实现经济社会又好又快发展。国民经济和社会发展的主要预期目标是：地区生产总值增长13%以上；全社会固定资产投资增长20%以上；招商引资实际到位资金增长20%以上；社会消费品零售总额增长15%；财政总收入增长20%以上；城镇居民人均可支配收入实际增长9%，农民人均纯收入实际增长9%；城镇登记失业率控制在4.5%以内；万元生产总值能耗下降4%；主要污染物排放总量减少2%；人口自然增长率控制在5‰以内。

实现上述目标，必须认真把握好以下几项基本要求：

第一，突出加快发展。始终突出加快发展主题，进一步解放思想，以思想的大解放推动经济社会的大发展。坚持以经济建设为中心，聚精会神搞建设、一心一意谋发展。加快对接和融入长株潭。继续大力实施工业强市、项目立市、开放活市、科教兴市、依法治市五大战略，强力推进新型工业化、新型城市化和社会主义新农村建设，创新招商引资、项目建设的思路和方法，大力优化经济发展环境，推动我市经济快速发展，奋力后发赶超。

第二，坚持科学发展。坚持又好又快，快中求好，着力把握发展规律、创新发展理念、转变发展方式、破解发展难题，提高发展质量和效益，不断增强发展的协调性。提升县域经济发展水平，促进区域协调发展。妥善处理经济发展与人口、资源、环境的关系，努力建设资源节约型和环境友好型社会，增强永续发展能力。

第三，大力改革开放。改革开放的力度越大，经济社会发展就越好越快。把改革作为推动经济社会发展的强大动力，深化政府机构、国有企业、财税、金融、价格、投资和社会管理体制改革，着力构建充满活力、富有效率、更加开放、有利于科学发展的体制机制。创新对外开放工作思路，扩大开放领域，优化开放结构，提高开放质量，提升开放型经济水平，开创对外开放新局面。

第四，构建和谐益阳。坚持以人为本，区别轻重缓急，加大投入力度，切实解决人民群众最关心、最直接、最现实的利益问题。千方百计扩大就业，提高城乡居民收入水平。加强社会管理，加快基础教育、社会保障、公共医疗服务、住房保障和文化服务体系等方面的建设，使全体人民共享改革发展的成果，确保社会和谐稳定，不断增强人民群众的幸福感。

三、2008年工作安排

（一）大力实施工业强市战略，加快推进新型工业化

大力发展园区工业。加强对园区发展的领导，强化考核评比，加强工作调度，搞好协调服务。抓好园区体制机制创新，打破行政区域界限，鼓励各地跨区域创办工业园区，跨园区落户引进的项目，实行税收分成，利益共享。加强园区产业建设，突出引进重大产业项目，壮大园区经济实力。加快园区基础设施建设，推进标准化厂房建设，合理确定项目用地规模和投资强度，促进园区集约发展，提高园区承载能力。要加快东扩，主动对接长沙。益阳高新区要大力发展装备制造、电子信息、新能源、新材料等产业，建设成为全省承接产业转移的重要基地。龙岭工业园、长春工业园要推进资源整合，与高新区产业互补、共同发展。桃江、安化、沅江、南县、大通湖经济开发区要发挥比较优势，强化产业招商，形成发展特色。沧水铺工业园和茅草街工业园要加快建设步伐，促进项目落户，尽快形成规模。切实抓好乡镇工业小区建设，引导乡镇工业小区科学规划、加大投入、规范管理，重点扶持一批基础较好、潜力较大的乡镇工业小区发展，努力打造一批工业“明星乡镇”。

加快企业创新步伐。推进企业制度创新、技术创新和管理创新。深化国有企业改革，加强对已改制企业的跟踪服务，扶助企业发展壮大。强化企业技术创新的主体地位，支持益阳橡塑集团国家级工程技术（研究）中心，艾华集团、益华水产等企业省级工程技术（研究）中心的建设。支持企业加快技术改造，突出抓好一批重大技改项目建设，坚持以电子信息技术改造提升传统产业，大力推进信息化与工业化的融合，促进产业优化升级。推行公司制法人治理结构，促进企业管理科学化、规范化。加强品牌建设，争创国家名牌产品、中国驰名商标和国家免检产品。平等保护物权，大力支持非公有制经济发展。

推进资源节约和节能减排。推进资源综合利用，积极争取资源型企业列入国家资源节约和环境保护中央预算内投资备选项目计划。大力发展循环经济，推行清洁生产。建立节能减排统计、监测、考核体系，严格执行节能减排问责制和一票否决制。加强规划项目引导，淘汰落后产能，推广创新技术，全面完成节能减排约束性指标。

培育壮大产业集群。认真实施产业发展规划，着力培育装备制造、食品加工、棉麻纺织、电力、造纸、建材、化工、冶金、新材料、电子信息等产业集群。集中力量抓大企业、大项目，下大力整合矿产、竹木、棉麻、茶叶等优势资源，严格准入制度，优化资源配置，扶持一批优势骨干企业发展壮大。紧紧围绕与核心企业配套、与长株潭产业协作和特色资源加工开发，加快发展一批资源加工型、劳动密集型和科技型中小企业，增强产业配套能力。

积极发展生产性服务业。认真实施中心城区现代物流业发展规划，加快物流园区建设，扶持有实力的物流企业发展。启动申报口岸城市的工作。引导金融机构优化信贷结构，改善信贷服务，扩大信贷投放，增加有效投入，加大对地方经济发展的支持力度。支持企业上市融资。引导保险市场健康有序发展。支持信用担保机构发展。规范发展法律、会计、审计、咨询、信息等现代服务业。

（二）坚持统筹城乡发展，扎实推进社会主义新农村建设

大力发展现代农业。坚持用工业化理念来抓现代农业，以新型工业化带动农业产业化。继续围绕优质稻米、林纸板材、水产畜禽、棉麻纺织、油蔬茶果五大农业产业链建设，调整和优化农业结构，大力发展大户和龙头企业。按照“一个产业抓一两个核心企业、培育一两个优势品牌”的思路，整合涉农财政资金，集中扶持发展一批具有高成长性的农业产业化龙头企业，培养一批市场占有率高的知名农业品牌。加强农产品基地建设，提高农业综合生产能力。稳定粮食播种面积和产量，大力发展无公害、绿色和有机农产品；调整养殖模式，推行规模养殖、健康养殖和小区养殖，重点建设2至3个年出栏生猪百万头县；大力培植农村新兴产业，促进高效园艺业、休闲农业和旅游农业发展。引导农民发展各种专业组织。完善基层农技服务体系，切实抓好动植物疫病防控，全面推行标准化生产，提高农产品质量安全水平。着力打造安化黑茶品牌，扶持益阳茶厂、白沙溪茶厂等一批优势黑茶企业做大做强。壮大林业产业，推进国家现代林业示范市建设。

切实改善农村生产生活条件。坚持把基础设施建设和社会事业发展的重点放在农村，大力加强农村公共设施建设。完成农村公路建设里程1400公里以上，建好一批农村客运站场。抓好防汛抗旱工作，加强山洪地质灾害防御，继续抓好洞庭湖综合治理，完善防灾减灾体系，搞好小流域治理和水库山塘除险加固，完成水利建设投入5.2亿元，实施各类水利工程6万处。努力提高农业机械化水平。推进农业综合开发，抓好乡村清洁工程、土地整理开发和血防工程等建设。加快农村改水、改厕、改厨、改栏步伐，推广农村清洁能源，新建沼气池1.2万个。抓好农村电网改造，改善农村通信条件。抓好社会主义新农村示范村（片）建设。

认真落实各项惠农政策。严格落实粮食最低收购价、各项农业补贴、水库移民后期扶持等惠农政策。认真执行减负政策，严格涉农价费监管，规范“一事一议”，取消共同生产费，切实减轻农民负担。积极发展农村小额信贷和农业保险。加强农业执法和农资市场管理，切实维护农民利益。

深化农村综合改革。坚持农村基本经营制度，按照依法自愿有偿的原则，引导农村土地经营权流转，促进土地适度规模经营。进一步深化乡镇机构改革、农村义务教育管理体制改革、县乡财政管理体制改革。积极推进并村并组，加大乡村债务化解力度。统筹推进农村金融体制、户籍制度、征地制度、集体林权和水权制度等改革，促进城乡经济社会协调发展。

（三）狠抓中心城区扩容提质，大力推进新型城市化

充分发挥区位交通优势和生态环境优势，实施“东接”战略，突出加强中心城区建设，大力推进新型城市化，以城市化带动工业化，以工业化促进城市化。

努力提升城市规划水平。按照“经济繁荣、环境优美、功能齐全、特色鲜明的环省会中心城市，现代化新型工业城市，宜居山水生态文化旅游城市”的定位，根据城市总体规划和土地利用总体规划，高标准编制好城市规划区控制性详细规划、修建性详细规划和专业规划。坚持城市规划例会审批、公开公示、听证等制度，严格规划审批，加强规划执法，确保各项规划得到执行。认真实施《益阳市会龙山公园控制性详细规划》及《资江南岸山体保护规划》。加强城中村、郊区、近郊区及“三线”地带的规划统筹管理，严厉查处违法违章建筑。

抓好城市建设与经营。加快益宁城际干道建设，实施桃花仑路、康富路改造工程，切实抓好益阳大道两厢的开发建设，全面启动资阳区旧城改造。开工建设资江四桥，继续抓好长春西路、十洲路、罗溪南路、团洲路、团圆路、云树路等工程建设。完成污水处理厂的配套管网建设和主要风景区、公园的配套设施建设，抓好主次干道两侧建筑物的亮化工程。以创建国家森林城市为目标，抓好城市绿化，开展生态文明单位创建活动。切实搞好城市经营，多方筹集建设资金。强化城市建设投资公司的投融资主体作用。高度垄断土地一级市场，依法清理闲置土地，严格实行以拍卖为主的招拍挂制度，坚持有计划出让“熟地”，实现土地收益最大化。规范出让广告经营权、出租车经营权、道路冠名权等特许经营权，推行市政设施的有偿使用。积极争取国家开发银行和其他商业银行的信贷支持，争取国债资金和有关专项资金。

切实加强城市管理。完善城市管理模式，加大城市管理投入，强化区级政府管理责任，充分发挥街道办事处、社区在城市管理中的作用，提高城市管理效能。严格依法管理城市，加强综合执法。加强宣传教育，提高市民素质，引导广大市民自觉维护公共秩序、爱护公共设施、保护城市环境。加强市容市貌整治，实行环境卫生管理的市场化运作，切实落实“门前三包”责任制，大力整治城市交通、市场和经营秩序，努力解决“脏乱差”问题，实现城市建设和管理的协调发展。

加强小城镇建设。完成县域村庄布局规划，启动新一轮重点镇规划修编。推进产业兴镇，努力将重点镇建设为经济重镇。切实抓好城镇经营，拓宽筹资渠道，加强基础设施建设和环境卫生管理，打造一批经济繁荣、特色鲜明的小城镇。

（四）突出抓好招商引资和项目建设，增强经济发展后劲

大力抓好招商引资。继续开展“招商引资年”活动。强化招商引资工作责任，规范招商引资政策，严格招商引资目标管理，优化招商引资环境，提高招商引资质量，形成主要领导带头招商，市县乡联动、部门参与的全员招商、全方位招商的工作格局。全年实际到位市域外资金220亿元，工业项目比重、园区项目入户率、项目履约率均达70%以上，引进1亿元以上重大工业项目30个以上。巩固对珠三角的招商成果，拓展面向长三角的招商空间。主动对接央企投资方向，突出引进战略投资者，突出工业园区招商，大力引进资源深加工和资金技术密集型项目。鼓励以商招商，支持本地民营企业通过增资扩股、技术改造、收购兼并等形式，引进战略合作伙伴，促进企业做大做强。

强力推进项目建设。严格目标管理，强化督查调度，大力促进项目实施，确保全年完成固定资产投资234亿元以上。完成托普竹麻产业建设、中冶集团钨业深加工、科力远三期、益华水产加工基地、柘溪电站扩容、株溪口电站等项目。加快益阳电厂二期、紫金矿业、省道204南茅复线、省道202线华茅公路、省道308线西姚公路、益芦航道、白竹洲电站等项目建设进度。抓好光伏产业基地建设，力争通过3年努力，将益阳打造成全国乃至亚洲最大的光伏产业基地。动工建设桃马公路。争取橡胶轮胎产业化工程、绕城高速、二广高速益阳段、杭瑞高速益阳段及其联络线、国道207线益阳段、省道308线黄柘段、益阳至横市二级公路等项目开工建设。重点推进桃花江核电站等项目的前期工作。继续加强项目库建设，开发储备一批大项目、好项目。

（五）加快发展第三产业，努力拓展城乡消费

大力发展文化旅游业。继续把旅游业作为支柱产业来培植，全力创建中国优秀旅游城市。全年接待国内外游客650万人次，旅游总收入突破35亿元。加强旅游与文化的融合，发展本土特色旅游。丰富生态观光旅游产品内涵，加快休闲度假旅游产品开发，有序推进民俗风情游、文化古迹游、宗教文化游、体育健身游。拓宽旅游融资渠道，抓好旅游资源开发，加强旅游基础设施建设，抓好高等级景区、高星级旅游饭店、旅游商品购物市场和游客咨询服务中心建设，集中力量打造茶马古道、桃花江竹海、会龙山佛教公园、梓山湖主题公园等旅游精品，加快洞市老街、梅山文化园、山乡巨变第一村、黄家湖生态园等景区景点建设，逐步开发胭脂湖、鱼形山、蚩尤故里、陶澍陵园、资江风光带等旅游资源。加强宣传促销，强化行业管理，提升旅游接待服务水平。

努力拓展城乡消费市场。合理引导住房、汽车、文化、健身等消费，培育新的消费热点，促进消费结构升级。深入推进“万村千乡市场工程”，建设高标准现代农家店300家以上，不断完善农村市场服务体系。支持益阳茶业市场、香港城商业中心、福中福商业城等现代流通企业发展，抓好高岭布匹市场建设。运用现代流通方式改造传统经营网络，加快传统零售业向连锁经营、传统仓储业向第三方物流业经营、传统集贸市场向超市化经营的新型业态转变。进一步加大整顿规范市场经济秩序的力度，完善行政执法、行业自律、舆论监督、群众参与相结合的市场监管体系，进一步优化消费环境。

积极扩大对外经济贸易。大力发展对外加工贸易，努力争取益阳列入国家加工贸易梯度转移重点承接地。优化出口产品结构，培育出口品牌。扶持橡胶机、科力远、益华水产等出口企业做大做强，培育出口龙头。引导有条件的企业到境外投资兴业。积极开展对外劳务输出。

（六）切实抓好财税工作，加快建立公共财政体系

强化财税征管。坚持依法治税，加强税法宣传，营造良好的税收征管环境。深化纳税评估，强化税源动态管理。突出工作重点，加强对重点地区、重点行业、重点税种、重点企业的监控，确保重点税源和主体税种收入稳定增长；坚持严征细管，加大对零散税源和易流失税收的征管力度，做到应收尽收。抓好非税收入的征收管理。规范税费减免行为。

加强支出管理。进一步优化支出结构，把财政资金更多地投入到推动科学发展和支持改善民生方面。进一步完善部门预算改革，全面推行国库集中收付制度改革，加强“收支两条线”管理，提高政府财政资金使用效益。加强财政专项资金管理。加强对高新区和大通湖区的财政监管。

（七）统筹发展各项社会事业，推动社会全面进步

继续实施科教兴市战略。充分发挥科技对经济发展的引领与支撑作用，加大对科技创新的融资和投入力度，大力推进科技创新。积极构建高新技术孵化平台和各类创业平台，推进科技创业。加强特色产业关键技术攻关与共性技术推广。支持有条件的企业申报国家中小企业创新基金。加强与高等院校、科研院所的合作，办好首届产学研对接与科技成果交易大会。坚持把教育放在优先发展的战略地位，加快建设教育强市。进一步推进素质教育，提高教育教学质量。全面启动义务教育学校标准化建设工程，完善“双线三级”保学控流工作机制，促进义务教育均衡发展。加快发展学前教育，大力发展职业教育，规范发展民办教育。深化教育人事制度改革，提高教师队伍素质。贯彻尊重劳动、尊重知识、尊重人才、尊重创造的方针，创新人才工作体制机制，培养、吸引和用好各类人才，激发各类人才创造活力和创业激情。

努力提高公共卫生和医疗服务水平。完善重大疾病防控体系，加大血吸虫病防治工作力度，提高突发公共卫生事件应急处置能力。抓好市中心医院外科大楼建设，发展城市社区卫生服务。继续推进乡镇卫生院建设，完善农村卫生服务网络，提高新型农村合作医疗参合率。加强卫生监督执法工作。规范医疗机构药品和医疗器械采购工作。开展爱国卫生运动。发展妇幼卫生事业。狠抓医德医风建设，提高医疗服务质量。

繁荣发展文化体育事业。加强公共文化服务体系建设，保障人民群众基本文化权益。加大社区和乡村文化设施建设投入力度，促进城乡、区域文化协调发展。繁荣文艺创作，丰富群众文化生活。深化文化体制改革，发展文化产业，增强文化发展活力。举办纪念周立波诞辰100周年活动。抓好对网吧市场、娱乐市场和非法出版物的专项治理。继续开展好“全民健身与奥运同行”系列活动，积极备战全省第十一届运动会，加强体育后备人才培养。

认真贯彻落实基本国策。扎实开展计划生育村级基础建设年活动，强化部门综合治理，狠抓流动人口计划生育管理，积极推行农村部分计划生育家庭奖励扶助和独生子女死亡伤残家庭扶助制度，加强出生缺陷干预，继续整治“两非”行为，严肃查处党员干部和社会公众人物的违法生育行为，努力提升全市人口和计划生育工作整体水平。加强基本农田保护和地质环境保护，严格建设用地审批管理，整顿和规范国土资源市场秩序。严格林地和森林采伐管理，加强矿产、水资源保护和合理开发。坚持环境保护与经济发展并重，执行建设项目环境影响第一审批权制度，巩固对造纸企业、涉锑企业和炼钒企业的污染整治成果，加强资江流域益阳段水资源保护工作，抓好重点区域和重点行业的环境综合整治，加快城镇环保基础设施建设步伐，加大农村生态保护力度，确保城乡环境安全。

全面发展其他各项社会事业。坚持正确的舆论导向，办好《益阳日报》。支持广播电视发展，加快启动数字电视工程，加大有线电视网络整合力度，继续推进广播电视“村村通”建设。加强食品和药品监管，确保食品和药品安全。改进审计方式，提高审计质量。继续做好档案、统计、民族、宗教、外事、侨务、对台、老龄、地方志、消防、气象、水文、无线电管理等工作。

（八）着力保障和改善民生，努力构建和谐社会

切实改善民生。围绕学有所教、劳有所得、病有所医、老有所养、住有所居的目标，继续为民办实事，加大改善民生的力度。健全统筹城乡的就业服务体系，完善市场就业机制，支持自主创业、自谋职业，鼓励灵活就业、弹性就业，促进以创业带动就业。落实就业扶持政策，完善就业援助制度，巩固零就业家庭就业援助成果。提高农民就业技能，促进农村劳动力转移就业。规范和协调劳动关系，依法维护劳动者权益。深化收入分配制度改革，增加城乡居民收入；着力提高低收入者收入，创造条件让更多群众拥有财产性收入。积极推进建立覆盖城乡居民的社会保障体系，进一步扩大养老、失业、工伤、生育保险覆盖面，全面启动城镇居民医疗保险，妥善解决困难企业职工参加医保问题。健全以城乡低保为基础的社会救助体系，全方位提高社会救助能力。抓好住房公积金扩面工作。加强价格监管，落实各项价格调控措施，努力保持价格基本稳定。高度重视安化库区建设，认真解决水库移民生产生活困难问题。加强扶贫开发。做好优抚安置工作。发展残疾人事业。继续关注关爱留守儿童。加快推进廉租住房和经济适用住房建设，切实解决无房户和低收入家庭住房困难。

加强社会主义精神文明建设。加强党的十七大精神的学习宣传贯彻工作。大力弘扬爱国主义、集体主义、社会主义思想，加强社会公德、职业道德、家庭美德、个人品德建设。加强和改进思想政治工作，抓好青少年思想道德教育。深入开展“迎奥运、讲文明、树新风”活动。巩固“创星”成果，抓好群众性精神文明创建工作。

加强民主法制建设。进一步增强民主法制观念，坚持和完善向人大及其常委会报告工作和向政协通报、协商工作的制度，广泛听取各民主党派、工商联和无党派人士的意见、批评和建议，认真办理人大代表建议和政协委员提案，自觉接受人大的法律监督、工作监督和政协的民主监督。积极支持工会、共青团、妇联等人民团体的工作。完善基层民主政治制度，继续加强城市社区建设，抓好农村社区建设试点，做好村民委员会第七次换届选举工作。大力推进依法治市，继续抓好“五五”普法教育。加强执法队伍建设，严格落实行政执法责任制，建立健全行政执法评议考核机制，充分发挥行政复议的作用，不断提高依法行政水平。

努力维护社会稳定。严格落实维稳工作责任制。强化社会治安综合治理，全面深入开展平安创建活动，推进社会治安防控体系建设，加强基层警务工作，深化严打整治斗争，保障人民群众生命财产安全。坚持安全发展，强化安全监管和安全执法，突出隐患整治，建立长效机制，防止重特大安全事故发生。完善突发事件应急管理机制。健全人民调解、司法调解、行政调解联动机制，突出抓好矛盾纠纷排查调处，加强和改进社会管理。畅通信访渠道，规范信访秩序，加强法律援助工作，维护群众的合法权益。

增强全民国防观念，加强国防动员建设，提高部队和民兵预备役建设质量。进一步增强人防意识，依法做好人防建设、管理工作。做好征兵和转业、退伍军人安置工作。争创“双拥”模范城，巩固军政、军民团结。

四、加强政府自身建设

大力提高执行力。执行力就是生产力，执行力就是发展力。提高执行意识，牢固树立正确的执行理念。牢固树立“夙兴夜寐、寝食难安”的为民理念，“不讲借口、勇于担当”的责任理念，“雷厉风行、快速高效”的效率理念，真正让执行成为自觉自发的行动。明确执行内容，提高执行效率。坚决贯彻执行党的路线、方针、政策，全面贯彻落实科学发展观；全面准确地执行法律、法规，严格按照法定权限和程序行使权力、履行职责，进一步规范行政行为，努力建设法治政府；严格执行上级的指示精神；认真执行市委的决议、决定，切实执行人大及其常委会的决议、决定，优质高效地执行政府的决策和工作部署，积极采纳人民政协的意见和建议。要讲大局、讲团结、讲配合，不搞“上有政策、下有对策”的不执行，不搞“利益优先、有所选择”的半执行；要主动配合，敢于负责，形成齐抓共管、整体联动的执行格局，确保政令畅通。强化保障机制，规范执行行为。建立和完善执行的动力机制，让想执行、能执行、会执行的公务员有目标、有方向、有奔头；建立和完善反馈机制，加强对执行情况的督促检查；建立和完善责任机制，做到人人有任务，人人有责任，人人有压力，切实把工作抓到位、抓到底、抓出成效。

努力提高服务水平。牢固树立以人为本的执政理念，强化政府公共服务职能，努力建设服务型政府。切实改进工作作风，不断提高服务水平。进一步深化行政审批制度改革，减少和规范行政审批，大力推行政务公开，增加政府工作透明度，不断优化经济发展环境，努力为基层、为群众、为企业提供优质服务。进一步改进服务方式，继续实行绩效管理和有错无为问责制，完善工作流程，简化办事程序，加快工作运转，提高服务效率。切实加强公务员队伍建设，努力打造一支政治坚定、业务过硬、作风优良的的公务员队伍，不断提高为人民服务的本领。

切实加强勤政廉政建设。坚持勤政为民。求真务实，真抓实干。勤于学习，勤于思考，勤于调查研究，勤于解决实际问题，把更多的时间和精力放在加快发展和改善民生上。加强督促检查，确保工作落实。深入贯彻落实中央关于党风廉政建设和反腐败斗争的部署和要求，扎实推进惩治和预防腐败体系建设，在坚决惩治腐败的同时，更加注重治本，更加注重预防，更加注重制度建设，拓展从源头上防治腐败工作领域。围绕规范行政权力运行，继续抓好商业贿赂专项治理工作，解决损害群众利益的突出问题。厉行节约，规范职务消费，降低行政成本。全面加强政风建设，自觉接受监督，把廉政建设和反腐败工作推向深入。

着力推进政府管理创新。推进政府管理体制改革，努力形成权责一致、分工合理、决策科学、执行顺畅、监督有力的行政管理体制。切实转变政府职能，加快推进政企分开、政资分开、政事分开、政府与市场中介组织分开。完善决策机制，建立社情民意反映制度、重大社会事项公示制度、社会听证制度和专家咨询制度，推进决策科学化、民主化。加快电子政务建设，高标准建好政府门户网站，完善政府新闻发布制度和信息公布制度，健全市长信箱、市长热线等政府与公众沟通机制，保障人民群众的知情权、表达权、监督权。精减文件、会议，改进文风、会风，不断提高行政效能。坚持不懈地推进政府管理创新，不断提升推动科学发展的能力，努力提高政府工作水平。

各位代表！我们正处在加快发展、实现后发赶超的关键时期，新的形势催人奋进，新的任务光荣艰巨。让我们高举中国特色社会主义伟大旗帜，以邓小平理论和“三个代表”重要思想为指导，紧密团结在以胡锦涛同志为总书记的党中央周围，在中共益阳市委的坚强领导下，深入贯彻落实科学发展观，同心同德，开拓进取，励精图治，奋发有为，为圆满完成今年的各项目标任务，实现经济社会又好又快发展而努力奋斗！
